# The Association of Aging-Related Polymorphisms with Susceptibility to Lung Cancer: A Case-Control Study in a Japanese Population

**DOI:** 10.31557/APJCP.2021.22.4.1279

**Published:** 2021-04

**Authors:** Hironobu Furuie, Masako Arimura-Omori, Naoki Hamada, Toyoshi Yanagihara, Chikako Kiyohara

**Affiliations:** 1 *Department of Preventive Medicine, Graduate School of Medical Sciences, Kyushu University, Fukuoka, Japan. *; 2 *Research Institute for Diseases of the Chest, Graduate School of Medical Sciences, Kyushu University, Fukuoka, Japan. *

**Keywords:** Aging, lung cancer, oligonucleotide/oligosaccharide-binding fold containing 1, telomerase reverse

## Abstract

**Background:**

Telomere length is associated with cancer as well as aging. Telomerase reverse transcriptase (*TERT*), telomere RNA component (*TERC*) and oligonucleotide/oligosaccharide-binding fold containing 1 (*OBFC1*) are known to be involved in telomere length regulation. The tumor suppressor p53 (TP53), which has been shown to interact with tumor protein p53-binding protein 1 (TP53BP1), is implicated in the response to telomere shortening and aging. Polymorphisms in the *TP53* and *TP53BP1* genes are associated with various types of cancer. The aim of this study is to evaluate the impact of aging-related polymorphisms on lung cancer risk.

**Materials and Methods:**

This case-control study consists of 462 lung cancer cases and 379 controls from Japan. We examined the effect of *TERT *rs2736100, *TERC *rs1881984*, OBFC1 *rs11191865*, TP53 *rs1042522 and *TP53BP1 *rs560191 on the risk of lung cancer using a Taq-Man real-time PCR assay. Unconditional logistic regression was used to assess the adjusted odds ratios (ORs) and 95% confidence intervals (CIs). Results: None of the main effects of any of the telomere-related polymorphisms were related to the risk of lung cancer. Similarly, none of the interactive effects of any of the telomere-related polymorphisms with smoking were associated with lung cancer risk. The significant multiplicative interaction between *TERT* rs2736100 and *TP53BP1* rs560191 was statistically significant (OR for interaction = 0.34, 95% CI = 0.14–0.84). The multiplicative interaction between *OBFC1 *rs11191865 and *TP53BP1 *rs560191 was also statistically significant (OR for interaction = 2.44, 95% CI = 1.02–5.87) but the OR for interaction was in the opposite direction.

**Conclusions:**

Our findings indicate that *TP53BP1 *rs560191 may predispose to lung cancer risk depending on the genotypes of telomere-related polymorphisms. Additional studies are warranted to confirm the findings suggested in the present study.

## Introduction

Lung cancer is a major cause of cancer-related death in developed countries and accounts for about one-quarter of all cancer deaths (Siegel et al., 2019). As tobacco smoking is an established risk factor for lung cancer, the number of lung cancer deaths continues to decline owing to tobacco control (Moolgavkar et al., 2012). However, not only environmental factors but also genetic differences are responsible for lung cancer susceptibility. A number of genetic polymorphisms influencing lung cancer risk have been identified over the last two decades (Wang et al., 2017).

As telomere length is highly correlated across tissues (Kimura et al., 2010; Wilson et al., 2008), lymphocyte telomere length is considered a valid surrogate for the measure of telomere length in specific tissues. Telomere shortening may cause decreased proliferative potential, thus triggering aging (Blackburn et al., 2006). Aging is a pivotal risk factor for cancer development. It has been reported that polymorphisms in both the telomerase reverse transcriptase (*TERT*) and telomerase RNA component (*TERC)* genes are associated with telomere length (Codd et al., 2010; Soerensen, 2012; Codd et al., 2013). Oligonucleotide/oligosaccharide-binding fold containing 1 (OBFC1, aka STN1) is also involved in telomere elongation (Wan et al., 2009). The tumor suppressor p53 (TP53), which has been shown to interact with tumor protein p53-binding protein 1 (TP53BP1), is implicated in the response to telomere shortening (Chin et al., 1999). TP53 has a role in regulating organismal aging (Tyner et al., 2002). TP53BP1, a DNA damage response factor, plays a pivotal role in maintaining genomic stability. Genomic instability has been implicated as the main causal factor in aging (Curtis, 1963). Therefore, *TERT, TERC, OBFC1, TP53* and *TP53BP1* can be considered as aging-related genes.

In this case-control study, we investigated the possible association between genetic polymorphisms in age-related genes (*TERT *rs2736100*, TERC* rs1881984*, OBFC1 *rs11191865*, TP53 *rs1042522 and *TP53BP1* rs560191) and lung cancer risk in a Japanese population. As smoking is the greatest risk factor for lung cancer, we investigated risk modification by the age-related polymorphisms in the association of smoking and lung cancer risk. We also examined the modifying effect of the *TP53* rs1042522 or *TP53BP1 *rs560191 genotypes, which were examined in a previous study (Kiyohara et al., 2010), on the association of any of the telomere-related polymorphisms with lung cancer risk.

## Materials and Methods

*Study subjects and data collection*

The 462 lung cancer patients were enrolled in Kyushu University Hospital (Research Institute for Diseases of the Chest, Kyushu University) and its collaborating hospitals. They were newly diagnosed with histologically confirmed primary lung cancer during the period from 1996 to 2008. Their cancers were histologically categorized into four types—adenocarcinoma, squamous cell carcinoma, small cell carcinoma and large cell carcinoma—according to the International Classification of Diseases for Oncology. All cases agreed to participate in this study. The 379 controls were inpatients who had not developed any type of malignancy, ischemic heart disease or chronic respiratory disease during the same period. Controls were not matched to cases either individually or in larger groups. The participation rate among the controls was 100%. All subjects were unrelated ethnic Japanese. Information on demographics and lifestyle factors including age, years of education, smoking and alcohol consumption was collected by self-administered questionnaire. This study was recognized by the Institutional Review Board of Kyushu University Hospital. We received written informed consent from all patients.

*Genetic analysis*

Genomic DNA was extracted from blood samples, and genotyping was conducted with blinding to case/control status. TaqMan^®^ SNP Genotyping Assays purchased from Applied Biosystems (Foster City, CA, USA) were used for the following [gene, single nucleotide polymorphism (SNP), assay ID]: *TERT*, rs2736100, C___1844009_10; *TERC*, rs1881984, C____176429_10; and *OBFC1*, rs11191865, C___2818536_10. The real-time PCR reaction conditions used are described in Arimura-Omori (2020). For quality control, we repeated assays on a random 5% of all samples, and the replicates were 100% concordant.

*Statistical analysis*

Comparisons of means and proportions were based on unpaired t-test and *χ*^2^ test, respectively. The distribution of polymorphisms among controls was compared with that expected from Hardy–Weinberg equilibrium (HWE) by Pearson’s *χ*^2^ test. Current smokers were those who had smoked or had stopped smoking less than one year prior to the date of diagnosis (cases) or the date of completion of the questionnaire (controls). Former smokers were those who had stopped smoking one or more years before the date of diagnosis (cases) or the date of completion of the questionnaire (controls). Never smokers were defined as those who had never smoked in their lifetime. Excessive drinkers were those who drank greater than 20 g/day alcohol because the Healthy Japan 21 guideline defines an appropriate volume of alcohol intake as 20 g/day (available at http://www.kenkounippon21.gr.jp/kenkounippon21/about/kakuron/index.html). Unconditional logistic regression was used to compute the odds ratios (ORs) and their 95% confidence intervals (CIs), with adjustments for several covariates (age, gender, smoking status, alcohol consumption and education). To test the interaction between genetic polymorphism and smoking or between SNPs, we performed a multiplicative interaction analysis. The multiplicative interaction was evaluated based on a likelihood ratio test, comparing the models with and without interaction terms. If the multiplicative interaction measure is statistically different from one, there is evidence of an interaction. In such a situation, the OR (OR_F1F2_) associated with both the factor F1 and the factor F2 is greater (less) than the product of the ORs (OR_F1_ and OR_F2_, greater than one) associated with each factor separately (OR_F1F2_ > OR_F1_ × OR_F2_ (OR_F1F2_ < OR_F1_ × OR_F2_)). To achieve adequate statistical power in multiplicative interaction analysis, subjects with at least one minor allele were grouped together. All statistical analyses were calculated using STATA version 15 (STATA Corporation, College Station, TX). All P-values were two-sided, with those less than 0.05 considered statistically signiﬁcant.

## Results

*Characteristics of study subjects*

We summarize the distributions of selected characteristics among subjects in Supplement and Supporting DATA 1 (SSD 1). Our analysis comprised 462 lung cancer patients as cases (242 with adenocarcinoma, 131 with squamous cell carcinoma, 69 with small cell carcinoma and 20 with large cell carcinoma). The lung cancer patients and controls were not matched with regard to age (P < 0.001) or sex ratio (P < 0.001). Case subjects tended to smoke (P < 0.001) and drink (P < 0.001) more than controls. In addition, the length of education years in cases was shorter than in controls (P < 0.001).

*Association between telomere-related polymorphisms and lung cancer risk*

Genotypic frequencies and associations between genetic polymorphisms of telomere-related genes and lung cancer risk are shown in SSD 2. Minor allele frequencies (MAFs) of *TERT *rs2736100, *TERC* rs1881984, and *OBFC1 *rs11191865 among the controls were 0.413, 0.360 and 0.332, respectively. Genotype distributions of these genes were consistent with HWE among controls. None of these polymorphisms were associated with the risk of lung cancer after adjustment for age, gender, smoking status, alcohol consumption and education.

*Interaction between telomere-related polymorphisms and cigarette smoking in relation to lung cancer risk*

Table 1 shows the interaction between telomere-related genetic polymorphism and cigarette smoking in relation to the risk of lung cancer. To achieve adequate statistical power, current and former smokers were bundled into ever smokers in the multiplicative interaction analysis. Similarly, subjects with at least one minor allele were grouped together. Subjects with at least one minor allele, who have lower risk, were used as the reference group. After adjustment for potential covariates, ever smokers had a higher risk of lung cancer compared with never smokers (OR = 3.17, 95% CI = 2.28–4.39; data not shown). Modifying effects between these three genetic polymorphisms and cigarette smoking were not found in relation to lung cancer risk.

*Interaction between telomere-related polymorphisms and our previously reported TP53 rs1042522 in relation to lung cancer risk*

In our previous study, *TP53 *rs1042522 was not significantly associated with lung cancer risk but the C allele was designated as a risk allele (Kiyohara et al., 2010). Based on these earlier findings, subjects with at least one risk (C) allele were bundled in one group for the present analysis. Following adjustment for potential confounders, there were no significant multiplicative interactions between telomere-related genetic polymorphisms and *TP53 *rs1042522 in relation to lung cancer risk (Table 2).

*Interaction between telomere-related polymorphisms and our previously reported TP53BP1 rs560191 in relation to lung cancer risk*

The GG genotype of *TP53BP1 *rs560191 was significantly associated with a decreased risk of lung cancer, as compared with the CC genotype (OR = 0.46, 95% CI = 0.29–0.74) (Kiyohara et al., 2010). According to the previous study (Kiyohara et al., 2010), subjects with at least one risk (C) allele of *TP53BP1 *rs560191 were bundled in one group. Interactions between telomere-related polymorphisms and* TP53BP1 *rs560191 in relation to lung cancer risk are shown in Table 3. In individuals with the GG genotype of *TP53BP1* rs560191, the TT genotype of *TERT *rs2736100 was significantly associated with an increased risk of lung cancer, compared with the TG and GG genotypes combined after adjustment for potential covariates (OR = 2.58, 95% CI = 1.12–5.94). As compared with the reference (at least one G allele of *TERT *rs2736100 and the GG genotype of *TP53BP1 *rs560191), lung cancer risk was approximately similar among subjects regardless of combination with *TERT *rs2736100 and *TP53BP1 *rs560191. As a result (ORF1F2 = 2.56/2.58*2.92 = 0.34), the multiplicative interaction between* TERT *rs2736100 and* TP53BP1 *rs560191 in relation to lung cancer risk was statistically significant (OR for interaction = 0.34, 95% CI = 0.14–0.84).

Subjects with the GG genotype of *OBFC1* rs11191865 and at least one C allele of *TP53BP1 *rs560191 (OR = 1.67, 95% CI = 0.93–2.99) presented a higher risk of lung cancer than those with at least one A allele of *OBFC1 *rs11191865 and at least one C allele of *TP53BP1 *rs560191 (OR = 1.37, 95% CI = 0.78–2.42), relative to the reference (subjects with at least one A allele of *OBFC1 rs11191865* and the GG genotype of *TP53BP1* rs560191). There was a significant multiplicative interaction between the *OBFC1* rs11191865 and *TP53BP1* rs560191 in relation to lung cancer risk (OR for interaction = 2.44 (1.67/0.50*1.37), 95% CI = 1.02–5.87).

Subjects with at least one C allele of *TP53BP1 *rs560191 had a significant 2-fold increased risk of lung cancer compared with those with at least one C allele of *TERC* rs1881984 and the GG genotype of *TP53BP1 *rs560191 (reference). A significant modifying effect between *TERC *rs1881984 and* TP53BP1 *rs560191 in relation to lung cancer risk was not observed (OR for interaction = 0.92, 95% CI = 0.39–2.21).

**Table 1:** Interaction between Telomere-Related Polymorphisms and Ccigarette Smoking in Relation to Lung Cancer Risk

Polymorphism		No. (%) of		OR (95% CI)			
	Smoking^a^	Cases	Controls	Crude	P-value	Adjusted^b^	P-value
*TERT *rs2736100							
TG+GG	Never	95 (20.6)	141 (37.2)	1.0 (Reference)		1.0 (Reference)	
TT	Never	58 (12.5)	68 (17.9)	1.27 (0.82–1.96)	0.29	1.11 (0.68–1.81)	0.67
TG+GG	Ever	195 (42.2)	101 (26.7)	2.87 (2.01–4.08)	<0.001	3.28 (2.19–4.92)	<0.001
TT	Ever	114 (24.7)	69 (18.2)	2.45 (1.65–3.64)	<0.001	3.32 (2.10–5.24)	<0.001
Multiplicative interaction				0.68 (0.38–1.21)	0.19	0.91 (0.47–1.74)	0.77
*TERC *rs1881984							
TC+CC	Never	78 (16.9)	121 (31.9)	1.0 (Reference)		1.0 (Reference)	
TT	Never	75 (16.2)	88 (23.2)	1.32 (0.87–2.01)	0.19	1.35 (0.85–2.17)	0.21
TC+CC	Ever	184 (39.8)	98 (25.9)	2.91 (2.00–4.24)	<0.001	3.94 (2.56–6.06)	<0.001
TT	Ever	125 (27.1)	72 (19.0)	2.69 (1.79–4.04)	<0.001	3.25 (2.05–5.15)	<0.001
Multiplicative interaction				0.70 (0.40–1.23)	0.22	0.61 (0.32–1.15)	0.13
*OBFC1 *rs11191865							
AG+AA	Never	78 (16.9)	114 (30.1)	1.0 (Reference)		1.0 (Reference)	
GG	Never	75 (16.2)	95 (25.0)	1.15 (0.76–1.75)	0.5	1.26 (0.79–2.02)	0.33
AG+AA	Ever	185 (40.1)	92 (24.3)	2.94 (2.01–4.30)	<0.001	3.71 (2.40–5.73)	<0.001
GG	Ever	124 (26.8)	78 (20.6)	2.32 (1.55–3.48)	<0.001	3.31 (2.09–5.26)	<0.001
Multiplicative interaction				0.69 (0.39–1.20)	0.19	0.71 (0.38–1.33)	0.28

OR, odds ratio; CI, confidence interval; ^a^Current and former smokers were combined (eversmokers); ^b^Adjusted for age, sex, alcohol consumption and education.

**Table 2:** Interaction between Telomere-Related Polymorphisms and *TP53 *rs1042522 in Relation to Lung Cancer Risk

Polymorphism		No. (%) of		OR (95% CI)			
	*TP53 *rs1042522	Cases	Controls	Crude	P-value	Adjusted^a^	P-value
*TERT *rs2736100							
TG+GG	GG	131 (28.4)	104 (27.5)	1.0 (Reference)		1.0 (Reference)	
TT	GG	75 (16.2)	57 (15.0)	1.04 (0.68–1.61)	0.84	1.11 (0.68–1.83)	0.66
TG+GG	CG+CC	159 (34.4)	138 (36.4)	0.91 (0.65–1.29)	0.61	0.95 (0.64–1.41)	0.79
TT	CG+CC	97 (21.0)	80 (21.1)	0.96 (0.65–1.42)	0.85	0.96 (0.61–1.51)	0.86
Multiplicative interaction				1.01 (0.57–1.78)	0.98	0.91 (0.47–1.75)	0.77
*TERC *rs1881984							
TC+CC	GG	122 (26.4)	90 (23.8)	1.0 (Reference)		1.0 (Reference)	
TT	GG	84 (18.2)	71 (18.7)	0.87 (0.58–1.32)	0.52	0.77 (0.48–1.25)	0.29
TC+CC	CG+CC	140 (30.3)	129 (34.0)	0.80 (0.56–1.15)	0.23	0.73 (0.48–1.11)	0.15
TT	CG+CC	116 (25.1)	89 (23.5)	0.96 (0.65–1.42)	0.84	0.95 (0.61–1.49)	0.82
Multiplicative interaction				1.38 (0.79–2.40)	0.26	1.68 (0.88–3.19)	0.11
*OBFC1 rs11191865*							
AG+AA	GG	125 (27.1)	84 (22.2)	1.0 (Reference)		1.0 (Reference)	
GG	GG	81 (17.5)	77 (20.3)	0.71 (0.47–1.07)	0.1	0.82 (0.51–1.33)	0.42
AG+AA	CG+CC	138 (29.9)	122 (32.2)	0.76 (0.53–1.10)	0.15	0.75 (0.49–1.15)	0.19
GG	CG+CC	118 (25.5)	96 (25.3)	0.83 (0.56–1.22)	0.33	0.95 (0.61–1.49)	0.84
Multiplicative interaction				1.54 (0.88–2.67)	0.13	1.54 (0.82–2.92)	0.18

OR, odds ratio; CI, confidence interval; ^a^Adjusted for age, sex, smoking status, alcohol consumption and education.

**Table 3:** Interaction between Telomere-Related Polymorphisms and *TP53BP1* rs560191 in Relation to Lung Cancer Risk

Polymorphism		No. (%) of		OR (95% CI)			
	*TP53BP1* rs560191	Cases	Controls	Crude	P-value	Adjusted^a^	P-value
*TERT* rs2736100							
TG+GG	GG	33 (7.2)	62 (16.4)	1.0 (Reference)		1.0 (Reference)	
TT	GG	24 (5.2)	19 (5.0)	2.37 (1.14–4.95)	0.02	2.58 (1.12–5.94)	0.03
TG+GG	CG+CC	257 (55.6)	180 (47.5)	2.68 (1.69–4.26)	<0.001	2.92 (1.71–4.99)	<0.001
TT	CG+CC	148 (32.0)	118 (31.1)	2.36 (1.45–3.83)	0.001	2.56 (1.46–4.48)	0.001
Multiplicative interaction				0.37 (0.17–0.82)	0.02	0.34 (0.14–0.84)	0.02
*TERC* rs1881984							
TC+CC	GG	34 (7.4)	52 (13.7)	1.0 (Reference)		1.0 (Reference)	
TT	GG	23 (5.0)	29 (7.6)	1.21 (0.60–2.44)	0.59	1.08 (0.49–2.40)	0.85
TC+CC	CG+CC	228 (49.3)	167 (44.1)	2.09 (1.30–3.36)	0.002	2.08 (1.21–3.57)	0.008
TT	CG+CC	177 (38.3)	131 (34.6)	2.07 (1.27–3.37)	0.004	2.07 (1.18–3.61)	0.01
Multiplicative interaction				0.82 (0.38–1.74)	0.6	0.92 (0.39–2.21)	0.86
*OBFC1 *rs11191865							
AG+AA	GG	40 (8.6)	38 (10.0)	1.0 (Reference)		1.0 (Reference)	
GG	GG	17 (3.7)	43 (11.4)	0.38 (0.18–0.77)	0.007	0.50 (0.22–1.11)	0.09
AG+AA	CG+CC	223 (48.3)	168 (44.3)	1.26 (0.77–2.05)	0.35	1.37 (0.78–2.42)	0.28
GG	CG+CC	182 (39.4)	130 (34.3)	1.33 (0.81–2.19)	0.26	1.67 (0.93–2.99)	0.09
Multiplicative interaction				2.81 (1.29–6.11)	0.009	2.44 (1.02–5.87)	0.046

OR, odds ratio; CI, confidence interval; ^a^Adjusted for age, sex, smoking status, alcohol consumption and education.

## Discussion

In this study, genotypes of telomere-related polymorphisms (*TERT* rs2736100*, TERC* rs1881984 and *OBFC1 *rs11191865) were determined in 462 lung cancer cases and 379 controls. The MAFs of *TERT* rs2736100*, TERC *rs1881984 and *OBFC1* rs11191865 among controls were 0.413, 0.360 and 0.332, respectively (SSD 2) and the genotypic distributions were consistent with HWE. The *MAF of *rs2736100 in our study (0.413) was somewhat higher than that in the HapMap-Japanese samples from dbSNP (0.376, available at https://www.ncbi.nlm.nih.gov/snp/?term=rs2736100) or those in previous reports in healthy Japanese (0.399 (Miki et al., 2010), 0.400 (Kamatani et al., 2010)). On the other hand, the *MAFs of *rs1881984 (0.360) and rs11191865 (0.332) in our study were similar to those of the HapMap-Japanese dbSNP (the corresponding figures of rs1881984 and rs11191865 were 0.390 (available at https://www.ncbi.nlm.nih.gov/projects/SNP/snp_ref.cgi?rs=1881984) and 0.343 (available at https://www.ncbi.nlm.nih.gov/projects/SNP/snp_ref.cgi?rs=11191865), respectively). The genotypic distributions of *TP53 *rs1042522 and *TP53BP1 *rs560191 were reported in our previous study (Kiyohara et al., 2010).

None of the main effects of any of the telomere-related polymorphisms were related to the risk of lung cancer. *TERT *rs2736100 has been relatively frequently examined in lung cancer studies, while* TERC *rs1881984 and *OBFC1 *rs11191865 have not yet been examined. The G allele of *TERT* rs2736100 is associated with longer telomere length (Codd et al., 2013). Some studies indicated an increased frequency of the G allele of* TERT *rs2736100 in lung cancer patients (Hsiung et al., 2010; Myneni et al., 2013; Wang et al., 2010), while other studies found no association (Chen et al., 2012; Li et al., 2016). It has been reported that telomere length differed according to histological type of lung cancer (Jang et al., 2008; Sanchez-Espiridion et al., 2014). Although we examined associations between the polymorphism and histological types, we found that the associations were similar across histological types (data not shown). So far, therefore, the associations between lung cancer risk and telomere length are inconclusive. We examined for the first time the association between *TERC *rs1881984 and *OBFC1 *rs11191865 in relation to lung cancer risk. A crucial point in some studies was the relatively small sample size, which did not allow a definitive statement to be made in regard to the role of telomere-related polymorphisms and lung cancer. Lack of reproducibility of genetic associations has been frequently observed and has been variously attributed to population stratification, phenotype differences, selection biases, genotyping errors, and other factors (Chanock et al., 2007; Khoury et al., 2007). At present, the best way of resolving these inconsistencies appears to be additional replication studies with larger sample sizes, although this may not be feasible for rare conditions or for associations identified in unique populations (Chanock et al., 2007).

It is widely accepted that lung cancer development requires environmental factors acting on a genetically predisposed individual. As smoking may induce oxidative stress and then irretrievable damage to the telomeric DNA (von Zglinicki, 2002), smoking may be associated with telomere length. Smokers (an established high risk population) with a genotype related to telomere length may be more susceptible to lung cancer than expected from the independent effects of the two (smoking and genetic) separate factors. In the present analysis, none of the modifying effects of any of the telomere-related polymorphisms with smoking were associated with lung cancer risk (Table 1).

Several lines of evidence (Bhara et al., 2019; Li et al., 2015; Liu et al., 2014; Kiyohara et al., 2010) have suggested that the polymorphism-polymorphism interaction may modify the risk for lung cancer. In the present study, significant multiplicative interactions between *TP53BP1 *rs560191 and either *TERT *rs2736100 (OR for interaction = 0.34, 95% CI = 0.14–0.84) or OBFC1 rs11191865 (OR for interaction = 2.44, 95% CI = 1.02–5.87) were observed (Table 3), although the interactions were in opposite directions. Lung cancer risk was approximately similar among subjects regardless of combination with *TERT *rs2736100 and *TP53BP1 *rs560191. In such a case, a negative interaction (the effect of the combined action of two predictors is less than the product of the individual effects) may naturally be anticipated. On the other hand, subjects with the GG genotype of *OBFC1 *rs11191865 and the GG genotype of *TP53BP1* rs560191 were unexpectedly at lower risk of lung cancer than the reference group (OR = 0.50, 95% CI = 0.22–1.11). In such a situation, a positive interaction (the effect of the combined action of two predictors is more than the product of the individual effects) may be logically expected. It remains unclear how either *TERT *rs2736100 or* OBFC1 *rs11191865 would interact with *TP53BP1 *rs560191 differently. Although *TP53* is well known to respond to telomere shortening (Chin et al., 1999), the mechanisms underlying the interactions between *TP53BP1 *rs560191 and the telomere-related polymorphisms are not known. The biological evidence for this polymorphism-polymorphism interaction needs further in-depth investigation. Nonetheless, to our knowledge this is the first study to examine the modifying effect between telomere-related polymorphisms and *TP53* rs1042522 in relation to lung cancer risk.

Several limitations of this study warrant mention. We cannot exclude chance as an explanation for the associations observed in this study. In addition, selection bias is a common problem of all case-control studies. That is, while controls should be representative of the source population from which the cases were drawn, when using hospital-based cases, it may be improbable to deﬁne the population from which the cases were drawn. On the other hand, hospital controls may be more appropriate because the study population can be defined as potential hospital users (dos santos Silva, 1999). The participation rates tend to be slightly higher in hospital-based case-control studies than in population-based case-control studies, and a high participation rate may reduce the possibility of selection bias (Morton et al., 2006). The participation rates of cases and controls were very high in the present study. However, our results should be interpreted carefully because the possibility of selection biases can not be completely excluded in case-control studies. Replication of findings from additional studies with larger sample sizes is very important before any causal inference can be drawn.

In conclusion, none of the three polymorphisms (*TERT *rs2736100*, TERC *rs1881984 and* OBFC1 *rs11191865) were associated with lung cancer risk. *TP53BP1 *rs560191 may predispose to lung cancer risk depending on the genotypes of either *TERT *rs2736100 or *OBFC1 *rs11191865. Future studies involving larger control and case populations will undoubtedly lead to a more thorough understanding of the roles of main and modifying effects of telomere-related polymorphisms in lung cancer development.

## Author Contribution Statement

C.K., N.H. and T.Y. contributed to conception and design of the study. C.K. contributed to the data collection. H.F. and M. A.-O. carried out the experimental work. H.F. and C.K. contributed to the data analysis and the manuscript preparation. All the authors critically read the initial manuscript, commented on all parts of the text, and approved the final version of the manuscript.

## Supplement and Supporting Data (SSD)

SSD 1. Selected characteristics of lung cancer cases and controls.

SSD 2. Association between telomere-related polymorphisms and lung cancer risk.
